# Implications of childhood adversity for women's perinatal sleep quality and depressive symptoms over time: A serial mediation model

**DOI:** 10.1111/jsr.14201

**Published:** 2024-03-26

**Authors:** Crystal Modde Epstein, Hellen Lustermans, Thomas P. McCoy, Roseriet Beijers, Esther M. Leerkes, Carolina de Weerth

**Affiliations:** ^1^ School of Nursing University of North Carolina Greensboro Greensboro North Carolina USA; ^2^ Donders Institute for Brain, Cognition and Behaviour Radboud University Medical Center Nijmegen The Netherlands; ^3^ Behavioural Science Institute Radboud University Nijmegen The Netherlands; ^4^ School of Health and Human Sciences University of North Carolina Greensboro Greensboro North Carolina USA

**Keywords:** childhood trauma, depression, postpartum, pregnant, sleep

## Abstract

This study sought to examine the effects of childhood adversity on the longitudinal associations between perinatal sleep quality and depressive symptoms, and to determine the prospective associations between these constructs over time. A cross‐lagged autoregressive model was used to examine the longitudinal association between sleep quality and depressive symptoms at four points during the perinatal period: 18 and 32 weeks of pregnancy, and 6 and 12 weeks postpartum. Longitudinal mediation models were used to examine whether sleep quality or depressive symptoms mediated the effects of childhood adversity on these symptoms. Most participants (86%) reported poor sleep quality during pregnancy. Significant cross‐lagged effects of depressive symptoms on subsequent sleep quality were observed during pregnancy and postpartum. Depressive symptoms significantly mediated the effects of childhood trauma on sleep quality during pregnancy, but sleep quality did not significantly mediate the effects of childhood trauma on depressive symptoms. While sleep quality and depressive symptoms tend to co‐occur, our analyses indicate that perinatal depressive symptoms work as the primary driver of sleep quality over time. Childhood adversity may have long‐reaching effects in women as it was associated with more depressive symptoms in the perinatal period, which in turn appeared to undermine sleep quality.

## 1 INTRODUCTION

Depression is one of the most prevalent and debilitating conditions during the perinatal period, affecting 20% of perinatal women (Yin et al., 2021). Sleep disturbances are also common during the perinatal period, with 90% of women reporting disruptions in their sleep (Christian et al., 2019). Childhood adversity, including experiences of abuse, neglect and family/peer dysfunction (FPD), is a significant risk factor for both perinatal depression and poor sleep (McWhorter et al., 2019; Racine et al., 2021). Women who experience childhood adversity get less sleep, and have greater trouble falling asleep and staying asleep (McWhorter et al., 2019). Additionally, women who experienced childhood adversity are more likely to experience chronic, recurrent depression that is more resistant to pharmacological (Nanni et al., 2012) and psychotherapeutic treatment approaches (Berry et al., 2021; Grote et al., 2012). Perinatal depressive symptoms and poor sleep quality tend to co‐occur (Chan et al., 2022). However, whether one set of symptoms drives the other over time is unclear. Therefore, this study focuses on examining the effects of childhood adversity on the prospective associations between perinatal sleep quality and depressive symptoms over time.

Childhood adversity has long‐lasting physiological effects on the stress response system, which can affect sleep quality (Ordway et al., 2021). Exposure to repeated stress, especially during childhood, leads to recalibration of the hypothalamic–pituitary–adrenal axis (Gunnar & Howland, 2022) and increased latent vulnerability to stress (Simon & Admon, 2023). Pregnant women exposed to childhood adversity have a substantially larger cortisol awakening response (Epstein et al., 2020, 2021), which has been shown to mediate the association between poor sleep during adolescence and risk for depression years later (Kuhlman et al., 2020).

During the perinatal period, there is conflicting evidence on whether sleep quality or depressive symptoms drive the prospective associations over time. Some evidence indicates that depressed mood is a more influential predictor of subsequent poor sleep quality (Foss et al., 2021), while other studies suggest that poor sleep quality is a more influential predictor of subsequent depressive symptoms (Bao et al., 2022; King et al., 2022). It is also possible that the association between sleep quality and depressive symptoms is reciprocal (Chan et al., 2022). To understand these complex associations, longitudinal methods are needed to parse out the prospective association of symptoms over time.

The following research questions are addressed in this study. (1) What are the relations between childhood adversity, sleep quality and depressive symptoms during pregnancy and postpartum? (2) What are the cross‐lagged relations between sleep quality and depressive symptoms over time? (3) (a) Do depressive symptoms mediate the association between childhood adversity and sleep quality over time? (b) Does sleep quality mediate the association between childhood adversity and depressive symptoms over time? Childhood adversity was conceptualized in two forms: childhood trauma (i.e. abuse and neglect); and FPD.

## 2 METHODS

### 2.1 Sample and procedures

**FIGURE 1:**
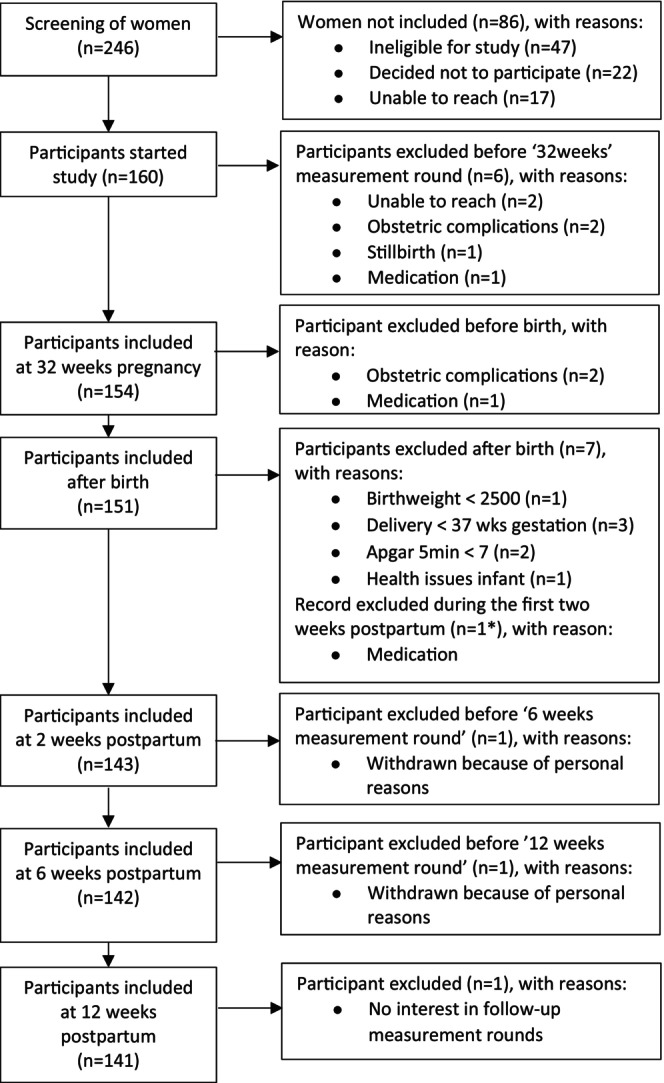
Flowchart of SMILEY participants.

Participants were part of the longitudinal SMILEY cohort (Study of Microbiota and Lifestyle in the Early Years), consisting of 160 mothers and their babies. Participants were recruited between December 2019 and April 2021 via the Baby & Child Research Centre's network of midwifery practices (The Netherlands) and social media. Inclusion criteria were: > 18 years old, mastery of the Dutch language, singleton pregnancy, pre‐pregnancy body mass index (BMI) ≤ 30 kg/m^2^, no severe obstetric complications, no current clinical diagnosis of a mental health disorder, no use of antidepressants, and no severe health issues. The study consisted of two prenatal (18 and 32 weeks) and two postnatal measurement rounds (6 weeks and 12 weeks; Figure 1). Participants received email invitations to complete online questionnaires at home and text message reminders when needed. Data were collected during the Covid‐19 pandemic lockdown, which did not affect the online data collection procedures. The study received ethical approval from Radboud University (#SW2017‐1303‐497). All participants provided written informed consent.

### 2.2 Measures

#### 2.2.1 Depression

The Edinburgh Postnatal Depression Scale (EPDS) is a 10‐item measure of depressive symptoms (Cox et al., 1987). Items are rated according to severity from zero (“no, not at all”) to three (“yes, quite often”). Scores range from 0 (none) to 30 (severe) and were analysed as a continuous variable in analyses. The Dutch version has favourable convergent validity with similar depressive symptom measures (Pop et al., 1992). Internal consistency was good (range ω = 0.85–0.90; *α* = 0.84–0.89). The EPDS was administered twice during pregnancy (18 and 32 weeks) and twice during postpartum (6 and 12 weeks).

#### 2.2.2 Sleep

The Pittsburgh Sleep Quality Index (PSQI) is an 18‐item measure of subjective sleep quality over the last month that includes seven subscales: subjective sleep quality; sleep latency; sleep duration; habitual sleep efficiency; sleep disturbances; use of sleeping medication; and daytime dysfunction (Buysse et al., 1989). The summed global sleep quality score ranges from 0 (no difficulties) to 21 (severe difficulties) and was analysed as a continuous variable. The Dutch PSQI has previously shown good internal consistency (*α* = 0.66) and test–retest reliability (*r* = 0.85, *p* < 0.001; Buysse et al., 1989; Henrich et al., 2023), but was lower than desired in the current study (range *α* = 0.37–0.51). Participants completed the PSQI at 18 and 32 weeks pregnancy.

The Groningen Sleep Quality Scale (GSQS) is a 14‐item scale reflecting subjective sleep quality from the previous night (Meijman et al., 1988). Because there can be substantial intra‐individual variation in postpartum sleep quality, we determined that a daily sleep measure (GSQS) would be more accurate in capturing patterns of postpartum sleep quality at 6 and 12 weeks compared with a monthly measure (PSQI). Like the PSQI, the GSQS measures sleep quality, duration, latency, efficiency and next‐day dysfunction, making it a comparable measure to use in models alongside the PSQI. Summed GSQS scores range from 0 to 14, with scores ≤ 2 indicating normal, refreshing sleep, and ≥ 6 indicating disturbed sleep. The GSQS has acceptable internal consistency reliability in Dutch samples (α = 0.82; Knufinke et al., 2018) and in the current study (range ω = 0.76–0.87; range α = 0.78–0.86). Continuous scores were used in analyses.

### 2.3 Childhood adversity

#### 2.3.1 Childhood trauma

The Childhood Trauma Questionnaire (CTQ) is a 25‐item measure of childhood experiences of abuse and neglect (Bernstein et al., 2003). Items are rated on a five‐point scale, ranging from 1 = “Never True” to 5 = “Very Often True” across five subscales: physical abuse; sexual abuse; emotional abuse; physical neglect; and emotional neglect. Total summed scores range from 25 to 125. The Dutch version of the CTQ has adequate reliability (α = 0.63–0.85) and validity for a five‐factor model (Thombs et al., 2009). Reliability via internal consistency in the current study was good (ω = 0.92, α = 0.91).

#### 2.3.2 Family/peer dysfunction

Family/peer dysfunction was measured using the Adverse Childhood Experiences International Questionnaire (ACE‐IQ) using the family and peer question groups (World Health Organization, 2020). In line with previous research, we measured family dysfunction as a distinct form of childhood adversity, separate from childhood trauma (i.e. abuse and neglect; Bussemakers et al., 2019). Items include alcohol or drug abuse in the household, incarcerated household members, someone chronically depressed, mentally ill, institutionalized, or suicidal, household violence, one or no parents, parental separation or divorce, and exposure to bullying. KR‐20 internal consistency was less than desired at 0.53. Each category was present (1) or absent (0), and summed for a total score ranging from zero to six.

### 2.4 Data analysis

**FIGURE 2:**
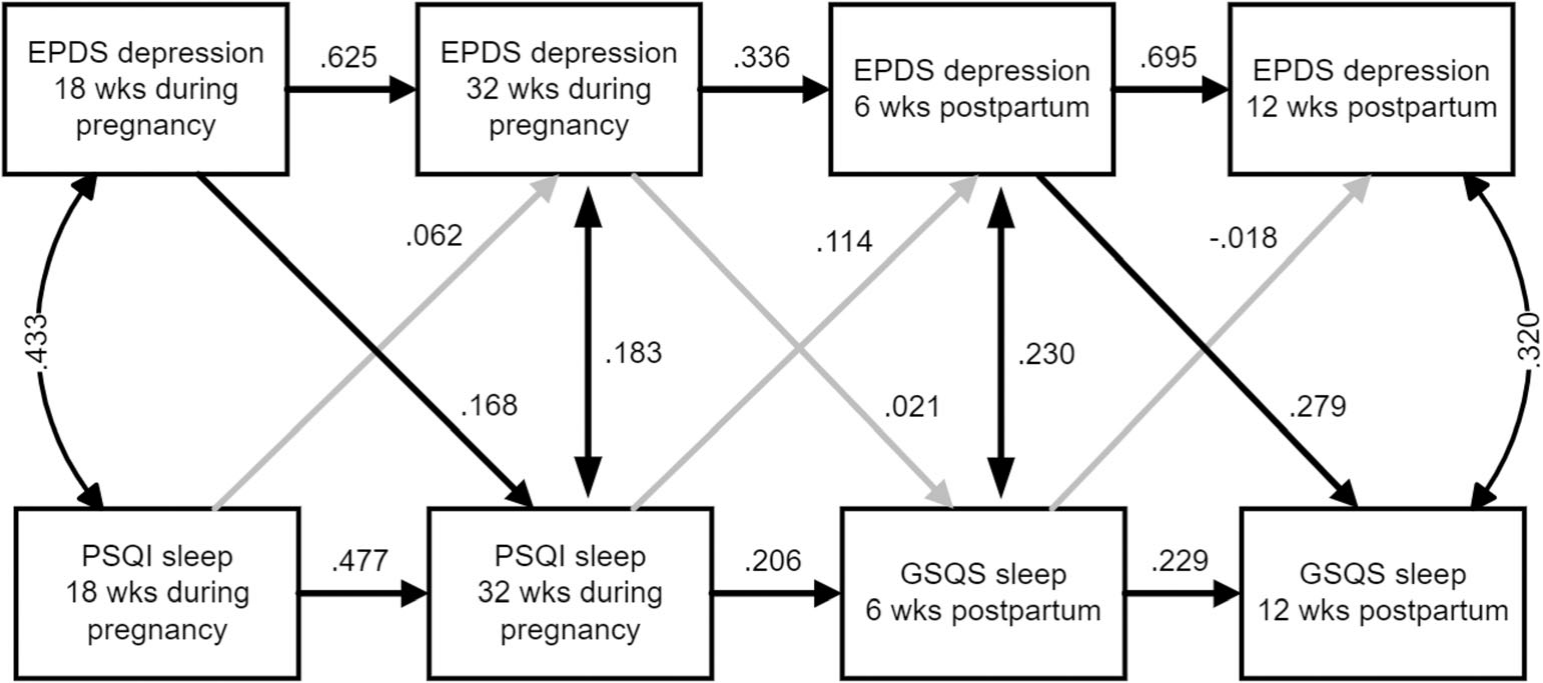
Path diagram of CLPM of depressive symptoms and sleep quality. Standardized estimates shown. Paths with significant direct effects (*p* < 0.05) shown in black arrows, while non‐significant effects shown in grey. CLPM, cross‐lagged panel model; EPDS, Edinburgh Postnatal Depression Scale; GSQS, Groningen Sleep Quality Scale; PSQI, Pittsburgh Sleep Quality Index.

Prior sample size calculations for longitudinal cross‐lagged panel model (CLPM) mediation analyses indicate a sample size of 150 is sufficient (≥ 87% power) to detect mediation with an effect size of 0.26 (Sedory, 2020), meaning our sample (*n* = 160) was adequately powered. Descriptive statistics were used to summarize sample characteristics (Table 1) and study measures (Table 2). Extreme outliers were defined as values beyond three times the interquartile range—below the 25th percentile or above the 75th percentile. Reliability via internal consistency was estimated using coefficients alpha and omega (Goodboy & Martin, 2020). Normality and presence of outliers were assessed with histograms, boxplots, normal *Q*‐*Q* plots and Shapiro–Wilk testing. Eigen analysis (Muller & Fetterman, 2002) was performed to examine multicollinearity, and no issues were found. For cross‐lagged effects, we used Orth et al.'s (2022) effect size guidelines of 0.03 (small effect), 0.07 (medium effect) and 0.12 (large effect). For mediation, 95% bias‐corrected bootstrap (bcb) confidence intervals (CIs) with 25,000 iterations were estimated. Indirect effects of interest were considered statistically significant if their 95% bcb CIs excluded zero. We examined Spearman correlations between study measures at their available time points after multiple imputation (MI) for missing data (Table 3) because of some extreme outliers in the study measures. For the MI, 1000 imputations were performed using predictive mean matching with fully conditional specification methods (Berglund & Heeringa, 2014). A four‐wave, cross‐lagged autoregressive model (CLPM) was used to examine the timing of the association between sleep quality and depressive symptoms over time (Figure 2). Modelling was performed using full information maximum likelihood (FIML) to adjust for missing data (Enders, 2010) in M*plus* v8.9 software (Muthén & Muthén, 2023). Additionally, maximum likelihood with robust standard errors and chi‐square test statistic estimation was performed, which is robust to non‐normality (Muthén & Muthén, 2023; Yuan & Bentler, 2000). Standardized direct and indirect effects not involving autoregressive paths were investigated to assess the mediation of effects of childhood trauma and FPD on sleep quality by depressive symptoms and on depressive symptoms by sleep quality, controlling for maternal age at 18 weeks of pregnancy, education level and pre‐pregnancy BMI. We also examined the five subtypes of childhood trauma by replacing the CTQ total score with each CTQ subscale in separate models. A two‐sided *p*‐value < 0.05 was considered statistically significant, and is reported with precision (95% CIs) and measures of effect size (standardized estimates, *R* ^2^).

**TABLE 1:** Demographic and health characteristics of the sample (*N* = 160)

Characteristic	*n* (%) or M ± SD (min, max)
Maternal age at 18 weeks gestation (years)	31.9 ± 3.6 (22, 42)
Missing	3 (1.9)
Country of origin	
The Netherlands	145 (90.6)
Other	13 (8.1)
Missing	2 (1.3)
Highest educational degree	
Low/medium^a^	21 (13.1)
High^b^	137 (85.7)
Missing	2 (1.3)
Currently have relationship with partner	
Yes	155 (96.9)
No	3 (1.9)
Missing	2 (1.3)
First child	
Yes	81 (50.6)
No	79 (49.4)
BMI (kg m^−2^)	23.0 ± 2.8 (17.3, 30.4)
Missing	1 (0.6)

^a^ Primary school, high‐school, MBO.

^b^ HBO, university. BMI, body mass index.

**TABLE 2:** Descriptive statistics for main study variables.

Measure	Time point			
	18 weeks pregnancy M [SD]	32 weeks pregnancy M [SD]	6 weeks postpartum M [SD]	12 weeks postpartum M [SD]
EPDS	4.58 [4.04] 10.1%^a^ 158 (98.8%)	5.31 [4.33] 9.7%^a^ 154 (96.3%)	5.24 [4.35] 12.1%^a^ 141 (88.1%)	4.85 [4.86] 11.5%^a^ 141 (88.1%)
PSQI	7.23 [2.13] 81.5%^b^ 157 (98.1%)	7.67 [2.42] 85.6%^b^ 153 (95.6%)		
GSQS			5.36 [2.94] 57.3%^c^ 131 (81.9%)	4.28 [3.48] 65.2%^c^ 141 (88.1%)
CTQ		34.6 [10.8] 148 (92.5%)		
FPD (ACE‐IQ)		1.07 [1.22] 149 (93.1%)		

*Note*: Number of missing values ranged from 2 (1.2%) to 29 (18.1%), while the number of non‐missing (%) is given in the third row for each measure.

^a^ Percentage of scores ≥ 11 cut‐off indicating possible depression. There were two extreme outliers in depressive scores: one at 32 weeks pregnancy and one at 6 weeks postpartum.

^b^ Percentage of scores > 5 indicating poor sleep quality during pregnancy. One extreme outlier.

^c^ Percentage of scores ≥ 6 indicating poor sleep quality during postpartum. ACE‐IQ, Adverse Childhood Experiences International Questionnaire; CTQ, Childhood Trauma Questionnaire; EPDS, Edinburgh Postnatal Depression Scale; FPD, family/peer dysfunction; GSQS, Groningen Sleep Quality Scale; PSQI, Pittsburgh Sleep Quality Index.

**TABLE 3:** Spearman correlations between all variables at all time points (*n* = 159)

Measure	1.	2.	3.	4.	5.	6.	7.	8.	9.	10.
1. EPDS‐18 weeks	1									
2. EPDS‐32 weeks	0.566**	1								
3. EPDS‐6 weeks	0.387**	0.338**	1							
4. EPDS‐12 weeks	0.457**	0.522**	0.570**	1						
5. PSQI‐18 weeks	0.402**	0.315**	0.062	0.103	1					
6. PSQI‐32 weeks	0.294**	0.335**	0.169*	0.286**	0.522**	1				
7. GSQS‐6 weeks	0.065	0.059	0.220**	0.094	0.207*	0.130	1			
8. GSQS‐12 weeks	0.192*	0.093	0.239**	0.360**	0.202*	0.295**	0.254**	1		
9. CTQ	0.246**	0.153	0.207*	0.173*	0.138	0.136	−0.030	0.069	1	
10. FPD	0.160	0.043	0.130	0.115	0.041	−0.002	0.129	0.088	0.554**	1

*Note*: Sample size is *n* = 159 after MI; pooled results presented.

Abbreviations: CTQ, Childhood Trauma Questionnaire; EPDS, Edinburgh Postnatal Depression Scale; FPD, family/peer dysfunction; GSQS, Groningen Sleep Quality Scale; PSQI, Pittsburgh Sleep Quality Index.

* *p* < 0.05.

** *p* < 0.01.

**FIGURE 3:**
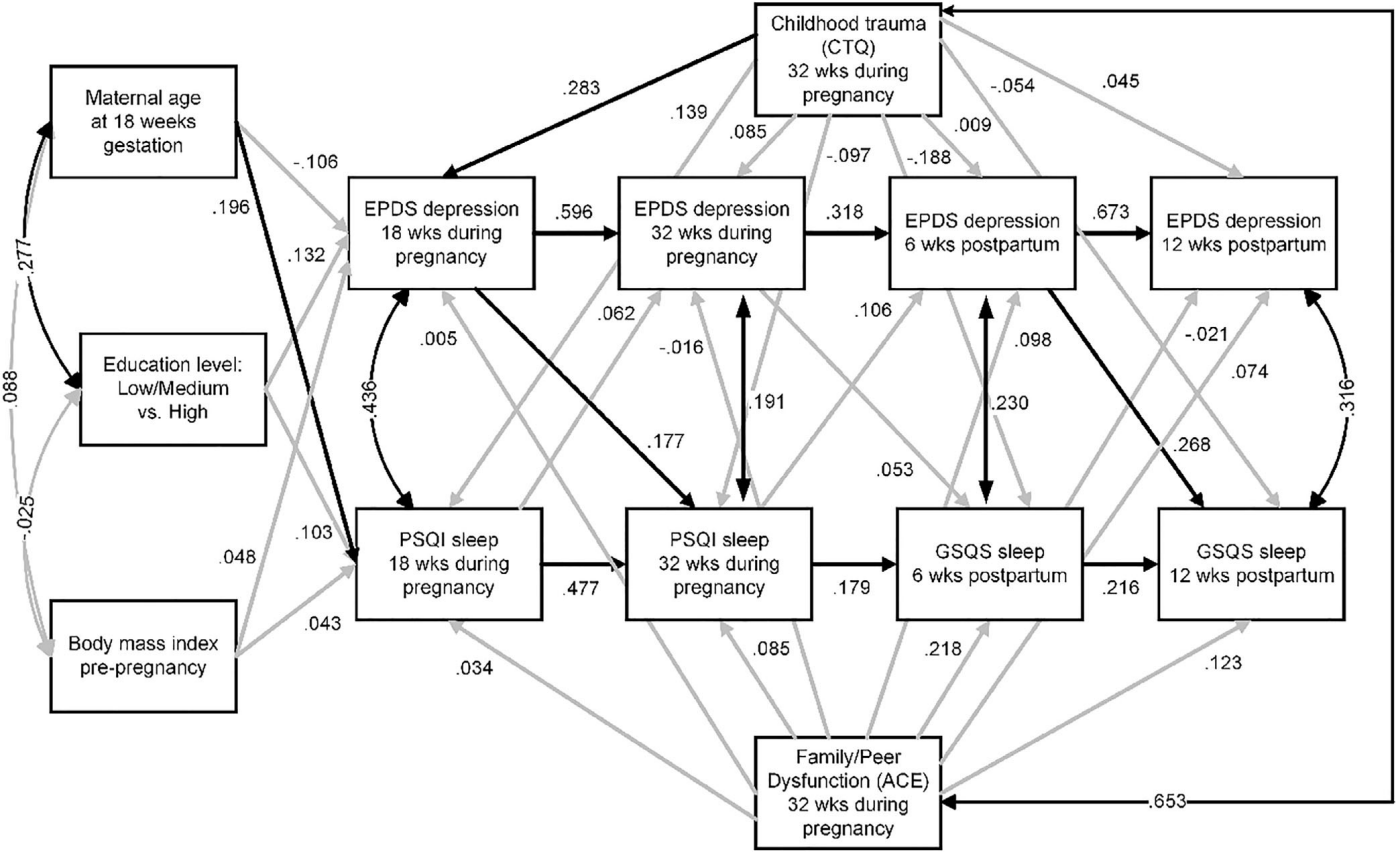
Path diagram of mediation CLPM of childhood trauma and FPD on depressive symptoms and sleep quality. Standardized estimates are shown with significant direct effects (*p* < 0.05) in black arrows, while non‐significant effects are shown in grey. CLPM, cross‐lagged panel model; CTQ, Childhood Trauma Questionnaire; EPDS, Edinburgh Postnatal Depression Scale; FPD, family/peer dysfunction; GSQS, Groningen Sleep Quality Scale; PSQI, Pittsburgh Sleep Quality Index.

Finally, because the EPDS includes an item about difficulty sleeping, sensitivity analyses were performed for correlations and modelling with this item removed. All conclusions about the CLPM in Figure 2 effects and in Figure 3 serial mediation for direct and indirect effects were exactly the same. We therefore present findings with the item retained so that our findings can be compared with previous studies using the EPDS. Furthermore, to address the limitation of using two different sleep scales, we performed sensitivity analyses to examine pregnancy and postpartum time periods separately in two‐wave CLPMs. None of the standardized direct effects was significantly different compared with the original four‐wave model; therefore, we present only the four‐wave model.

## 3 RESULTS

Most of the sample were Dutch (91%), had a university degree (54%), and were currently in a relationship (97%; Table 1). The percentage of the sample who scored ≥ 11 for depressive symptoms ranged from 9.7% to 12.1% (Table 2). Most participants reported poor sleep quality during pregnancy (PSQI scores > 5, 82%–86%) and postpartum (GSQS scores ≥ 6, 57%–65%).

There were two extreme outliers in depressive scores: one at 32 weeks of pregnancy and one at 6 weeks postpartum, so therefore Spearman correlations were estimated among study measures after MIs (Table 3). Childhood trauma and FPD measures were moderately correlated (*r*_s_ = 0.55). Prenatal depression and PSQI sleep quality were modestly correlated (range *r*_s_ = 0.34–0.40), while postnatal depressive symptoms were correlated with GSQS sleep quality to a lesser degree (range *r*_s_ = 0.22–0.36). Childhood trauma (CTQ) was significantly correlated with depressive symptoms at 18 weeks pregnancy (*r*_s_ = 0.25), 6 weeks postpartum (*r*_s_ = 0.21) and 12 weeks postpartum (*r*_s_ = 0.17), but was not correlated with sleep quality at any time point. FPD was not correlated with sleep quality or depressive symptoms.

Figure 2 presents the path diagram of the estimated CLPM of depressive symptoms and sleep quality measures. There were two significant cross‐lagged relations: depressive symptoms at 18 weeks during pregnancy on sleep quality at 32 weeks during pregnancy (*b* = 0.17, *p* = 0.02); and depressive symptoms at 6 weeks postpartum on sleep quality at 12 weeks postpartum (*b* = 0.28, *p* < 0.01).

Results from mediation modelling are presented in Table 4 and Figure 3. The *R* ^2^ statistics ranged from 0.07 to 0.48 for dependent variables. There were significant indirect effects of childhood trauma (CTQ) on sleep quality at 32 weeks during pregnancy via depressive symptoms at 18 weeks during pregnancy (*b* = 0.05, 95% bcb CI = [0.01, 0.13]). There were no statistically significant indirect effects for other time points or for FPD. Thus, we conclude that depressive symptoms mediate childhood trauma on sleep quality during pregnancy. However, sleep quality did not significantly mediate childhood trauma or FPD effects on depressive symptoms during or after pregnancy.

**TABLE 4:** Indirect effects of childhood trauma (CTQ) and FPD on depressive symptoms and sleep quality (*n* = 159)

Dependent	*R* ^2^	Independent	Direct	Indirect	
				CTQ	FPD
EPDS‐18 weeks	0.120	CTQ	**0.28 (0.09, 0.50)**	–	–
		FPD	0.01 (−0.21, 0.22)	–	–
PSQI‐18 weeks	0.067	CTQ	0.14 (−0.10, 0.38)	–	–
		FPD	0.03 (−0.18, 0.28)	–	–
EPDS‐32 weeks	0.424	CTQ	0.09 (−0.12, 0.29)	0.01 (−0.01, 0.06)	–
		FPD	−0.02 (−0.20, 0.16)	–	< 0.01 (−0.01, 0.04)
PSQI‐32 weeks	0.329	CTQ	−0.10 (−0.31, 0.12)	**0.05 (0.01, 0.13)**	–
		FPD	0.09 (−0.09, 0.29)	–	< 0.01 (−0.04, 0.05)
EPDS‐6 weeks	0.163	CTQ	0.01 (−0.22, 0.24)	−0.01 (−0.07, 0.01)	–
		FPD	0.10 (−0.11, 0.28)	–	0.01 (−0.01, 0.07)
GSQS‐6 weeks	0.073	CTQ	−0.19 (−0.39, 0.01)	< 0.01 (−0.01, 0.06)	–
		FPD	**0.22 (0.01, 0.42)**	–	< −0.01 (−0.03, 0.02)
EPDS‐12 weeks	0.483	CTQ	0.05 (−0.14, 0.25)	< 0.01 (−0.02, 0.08)	–
		FPD	0.07 (−0.12, 0.26)	–	< −0.01 (−0.06, 0.03)
GSQS‐12 weeks	0.173	CTQ	−0.05 (−0.23, 0.16)	< 0.01 (−0.07, 0.07)	–
		FPD	0.12 (−0.08, 0.35)	–	0.03 (−0.02, 0.10)

*Note*: Only direct and indirect effects for CTQ and FPD are shown. Cross‐lagged and autoregressive direct effects shown in Figure 2. FIML estimation for missing data was used; One case was missing all data. Numbers are standardized estimates and 95% bcb CIs after 25,000 draws. Bold values indicate CIs that do not include zero, indicating significant effects.

Abbreviations: CTQ, Childhood Trauma Questionnaire; EPDS, Edinburgh Postnatal Depression Scale; FPD, family/peer dysfunction; GSQS, Groningen Sleep Quality Scale; PSQI, Pittsburgh Sleep Quality Index.

Analysis of CTQ subscales revealed that the cross‐lagged results were mostly consistent with the CTQ total score results in Figure 3. For all subscales, the same cross‐lagged effects were observed, with depressive symptoms being associated with subsequent poor sleep quality during pregnancy and postpartum. The one exception was sexual abuse, for which only the postpartum cross‐lagged effects were significant. Similar to Figure 3, the effects of emotional and sexual abuse were significantly mediated by depressive symptoms on sleep quality at 18 weeks of pregnancy; however, emotional neglect, physical neglect and physical abuse were not significantly mediated by depressive symptoms at any time point.

## 4 DISCUSSION

Using four waves of longitudinal data, we examined cross‐lagged pathways to determine whether perinatal sleep quality or depressive symptoms drive the prospective associations between these constructs over time, mediating the effects of childhood adversity. There are two main findings. First, cross‐lagged findings indicate that, in this sample, depressive symptoms were significantly predictive of subsequent poor sleep quality during pregnancy and postpartum but not vice versa. Secondly, perinatal depressive symptoms mediated childhood trauma on perinatal sleep quality, but perinatal sleep quality did not significantly mediate the effects of childhood trauma on perinatal depressive symptoms. It is important to note, however, that such effects may be small and below what this study was powered to detect.

In this sample, we found that neither childhood trauma nor FPD was significantly associated with sleep quality at any point during pregnancy or postpartum. This differs from a recent systematic review reporting robust associations between childhood trauma and sleep disturbances in a non‐perinatal sample (Brown et al., 2022). The relationship between childhood trauma and sleep quality could be weakened during the perinatal period due to a greater influence of physical discomfort during pregnancy and care needs of the infant during postpartum.

Family/peer dysfunction was not significantly associated with sleep quality or depressive symptoms at any time point, suggesting that childhood trauma (i.e. abuse and neglect) is a more potent risk factor for depressive symptoms relative to FPD. This is consistent with non‐perinatal community samples in which child maltreatment was more strongly associated with depressive symptoms in adulthood than peer victimization or FPD (Sayyah et al., 2022).

The significant lagged association between depressive symptoms and later sleep quality in our study is consistent with another previous research study in a perinatal sample (Foss et al., 2021). In our sample, the lagged relationship was significant during pregnancy (18–32 weeks) and postpartum (6–12 weeks postpartum), but not between late pregnancy (32 weeks) and early postpartum (6 weeks). This could be related to characteristically poor sleep quality for most mothers during this period due to newborns waking frequently during the night, resulting in the erosion of individual differences that would otherwise be apparent over longer periods.

Prior research indicates that sleep disturbances, notably insomnia and sleep loss, affect multiple physiological systems, which play a critical role in the development of subsequent mood disorders (Palagini et al., 2019). Contrary to expectations, our findings diverged from this established evidence, which could be interpreted in a couple of ways. First, our study did not account for specific sleep disturbances such as insomnia or reduced sleep duration. Previous research has identified insomnia as a factor that nearly triples the risk of future depression (Hertenstein et al., 2019). Although poor sleep quality is common among perinatal women, only a minority (38%) suffer from insomnia or reduced sleep duration (Sedov et al., 2021).

Another consideration is the potential impact of the time lag between poor sleep quality and the manifestation of depressive symptoms. In our study, there was a 14‐week time lag between pregnancy time points (week 18 to week 32) and pregnancy (32 weeks) to postpartum (6 weeks). This is important because the strength and directional timing of the association between depressive symptoms and sleep quality may vary depending on the length of lag. Some longitudinal studies have examined time lags as long as 1 or 2 years (Hayley et al., 2015; Marino et al., 2022), whereas other studies have examined lags as short as 1 day (Nota et al., 2020; Triantafillou et al., 2019). For example, in a 7‐year longitudinal study using time lags of 1–2 years, there were significant associations from depressive symptoms to later sleep quality, but not from sleep quality to later depressive symptoms (Hayley et al., 2015). With a shorter lag (i.e. days), Nota et al. (2020) examined the day‐to‐day cross‐lagged relationships between sleep quality and psychopathological symptoms among non‐pregnant individuals receiving cognitive behavioural therapy. They reported significant lagged associations between poor sleep quality the previous night and greater psychopathology symptoms the next day, but not vice versa (Nota et al., 2020). Thus, the length of time lag may moderate the cross‐lagged associations between sleep quality and depressive symptoms, such that poor sleep quality is associated with depressive symptoms over shorter time lags (i.e. days), while depressive symptoms are associated with sleep quality over longer time lags (i.e. months, years). This hypothesis should be tested in future research.

We found that sleep quality did not significantly mediate the effects of childhood adversity on depressive symptoms during pregnancy. This is unexpected given theory and evidence indicating that sleep is a mechanistic mediator of early life adversity on later mental health outcomes (Fuligni et al., 2021). Using a similar mediated cross‐lagged model with a sample of pregnant adolescents, Foss et al. (2021) likewise reported that only perinatal distress significantly mediated the effects of childhood trauma on sleep quality and not vice versa. These findings contradict other studies using other types of statistical models (e.g. parallel process growth models), indicating that sleep quality drives the persistence of depressive symptoms over time (Tomfohr‐Madsen et al., 2022). Another consideration that may affect the findings is the level of depressive symptoms at baseline. Felder et al. (2023) reported that a single‐item measure of sleep disturbance among non‐depressed women in the second and third trimesters was associated with a threefold increased risk for postpartum depressive symptoms. Thus, poor sleep quality may be an important predictor of future perinatal depressive symptoms, especially among women who are not depressed.

### 4.1 Strengths and limitations

Strengths include the use of four waves of longitudinal data during the perinatal period. Generalizability is limited due to the relatively homogenous community sample of healthy, highly educated Dutch women. Limitations include reliance on subjectively reported global sleep quality scores rather than objective sleep measures (e.g. actigraphy), lack of control for day of the week, work shift (although note that all postpartum women were on maternity leave), and other postpartum factors (e.g. bedsharing and breastfeeding). Our study did not consider other sleep‐related conditions, such as insomnia, short sleep duration, restless legs syndrome or sleep apnea, all of which may have affected the results.

We used different sleep measures pre‐ and postnatally; however, sensitivity analyses indicated that this limitation had no significant effect on our main conclusions. Some measures had lower than desired internal consistency, such as the PSQI and ACE. Here, the sleep efficiency component of the PSQI underwhelmed in item analysis, which might have been due to unusually low sleep efficiency scores (88% at 18 weeks, and 87% at 32 weeks pregnancy), leading to small variance and covariance. Childhood adversity was measured retrospectively in the third trimester, but the CTQ is considered a relatively reliable retrospective measure in perinatal samples (Cammack et al., 2016). We did not use a diagnostic measure of depression and did not control for trauma‐related psychopathology, such as post‐traumatic stress disorder. Finally, the extent to which the COVID‐19 pandemic affected the results is unknown.

It is also important to note that self‐report measures of sleep quality are subject to various biases, including recall bias and mood‐congruent bias. However, previous studies using both objective (i.e. actigraphy) and subjective self‐report sleep measures have found that both subjective and objective sleep measures are significantly associated with childhood adversity (Sheehan et al., 2020). In samples of perinatal women, only subjective self‐reports of sleep quality, but not actigraphy, were associated with perinatal depression (Stremler et al., 2020; Volkovich et al., 2016). Interestingly, interaction analyses indicated a higher concordance between subjective/objective sleep measures among women with higher depression scores, suggesting low levels of mood‐congruent bias in self‐reported sleep quality among perinatal women (Volkovich et al., 2016).

### 4.2 Implications

The findings of this study are clinically relevant because significant associations between childhood adversity, depressive symptoms and sleep quality were present among a relatively healthy, low‐risk community sample of perinatal women. This is important because perinatal sleep quality and depressive symptoms contribute to higher perceptions of parenting stress, and decrease women's capacity to provide sensitive and quality parenting (Bai et al., 2020; Galbally et al., 2019). Social support buffers the harmful effects of poor sleep quality on both depressive symptoms and parenting stress (Camisasca et al., 2021); however, support alone tends to be less effective and less available for women with a trauma history and high depressive symptoms (Galbally et al., 2019). Trauma‐informed relational interventions that focus on bolstering protective factors are effective in improving mental health, maternal representations and parenting outcomes (Rosenblum et al., 2018; Rosenblum et al., 2017). Obstetric and family providers need to be trained in these approaches in order to increase the availability of these interventions (Erickson et al., 2019). Alternatively, psychology professionals could be integrated into perinatal care in order to screen for maternal mental health symptoms, carry out preventive interventions and treatments, and refer to more specialized care when needed.

## 5 CONCLUSION

Depressive symptoms and poor sleep quality are among the most common symptoms experienced by perinatal women. These symptoms can be especially severe and prevalent among women with a history of childhood adversity. While sleep quality and depressive symptoms tend to co‐occur, our analyses indicate that perinatal depressive symptoms are a stronger driver of sleep quality over time, especially from the second to the third trimester and from early to late postpartum. Furthermore, depressive symptoms significantly mediated the effects of childhood adversity on poor sleep quality, but not vice versa.

Regardless, it is important that perinatal women be screened for both sleep difficulties and depressive symptoms, and referred for evidence‐based treatments accordingly. Such preventative interventions may be critical for promoting maternal well‐being and interrupting intergenerational effects of maternal childhood adversity on long‐term childhood health outcomes.

## AUTHOR CONTRIBUTIONS

**Crystal Modde Epstein:** Conceptualization; writing – original draft; methodology. **Hellen Lustermans:** Writing – original draft; project administration; data curation. **Thomas P. McCoy:** Methodology; formal analysis; writing – original draft. **Roseriet Beijers:** Writing – review and editing; project administration. **Esther M. Leerkes:** Writing – review and editing; conceptualization; methodology. **Carolina de Weerth:** Conceptualization; funding acquisition; methodology; writing – review and editing; supervision.

## FUNDING INFORMATION

This research was supported by a Netherlands Organization for Scientific Research grant (016.Vici.185.038, to C. de Weerth).

## CONFLICT OF INTEREST STATEMENT

The authors declare no conflicts of interest or financial disclosures.

## Data Availability

Research data are not shared.
